# Complete genome sequence of multi-drug-resistant *Salmonella enterica* subsp. *enterica* serovar Typhimurium strain Bnaya isolated from a dairy calf in Israel

**DOI:** 10.1128/MRA.00351-23

**Published:** 2023-10-03

**Authors:** Chagai Davidovich, Aya Awwad, Marcelo Fleker, Limor Weisblit, Eddie Cytryn, Shlomo E. Blum

**Affiliations:** 1 Water and Environmental Sciences, Institute of Soil, Agricultural Research Organization, Rishon-Lezion, Israel; 2 Department of Agroecology and Plant Health, The Robert H. Smith Faculty of Agriculture, Food and Environment, The Hebrew University of Jerusalem, Rehovot, Israel; 3 Department of Bacteriology and Mycology, Kimron Veterinary Institute, Bet Dagan, Israel; 4 Department of Animal Sciences, The Robert H. Smith Faculty of Agriculture, Food and Environment, The Hebrew University of Jerusalem, Rehovot, Israel; University of Maryland School of Medicine, Baltimore, Maryland, USA

**Keywords:** *Salmonella*, Typhimurium, dairy, cattle, genomes, nanopore sequencing, antibiotic resistance

## Abstract

This manuscript reports the complete genome sequence of a *Salmonella enterica* subsp. *enterica* serovar Typhimurium strain (designated “Bnaya”), isolated from a dead dairy calf with severe diarrhea in Israel. The isolate exhibited multi-drug resistance, which is highly unusual in bovine *Salmonella* spp. in Israel, prompting further investigation.

## ANNOUNCEMENT

Serovar Typhimurium is a major non-host-adapted and zoonotic serovar of *Salmonella enterica* subsp. *enterica*, causing enteritis and septicemia in newborn calves and human foodborne enteric disease (1). We report the whole-genome sequencing of a *S*. Typhimurium strain isolated from mesenteric lymph nodes of a 1-week-old dead dairy calf, following severe diarrhea, in the agricultural village of Bnaya, Israel.

Samples were processed as previously described (2). Suspected colonies were identified as *Salmonella* spp. by MALDI-TOF-MS (score >2.0) with an Autoflex system, following the direct colony method (Bruker Daltonics, Billerica, MA, USA) (3), and assigned to serogroup 4 (former B) by slide agglutination (SSI Diagnostics, Hillerod, Denmark) (4). Three isolated colonies were selected for antibiogram by disk diffusion (5). The isolate was resistant to ampicillin, cephalotin, cefotaxime, gentamycin, ticarcillin, enrofloxacin, cefuroxime, sulfamethoxazole-trimethoprim, and florfenicol, and sensitive to amikacin, amoxicillin-clavulanic acid, and imipenem (Oxoid, Wesel, Germany). Because multi-drug resistance is highly unusual in bovine *Salmonella* spp. in Israel and due to public health concerns, we sequenced the genome of this strain, which was designated “Bnaya.”

A single colony was streaked on blood agar (5% sheep-blood-enriched tryptone agar [Merck, Darmstadt, Germany]) and grown overnight at 37°C. DNA was extracted using the DNeasy Blood & Tissue Kit (Qiagen, Hilden, Germany), following the protocol for Gram-negative bacteria. A DNA library was prepared with the Rapid Barcoding Sequencing Kit (SQK-RBK004; ONT, Oxford, UK), and sequencing was performed on a Flongle flow cell (R9.4.1) for 24 h, with an output of 82.3 k reads and an N50 of 9.24 kb. Basecalling was performed with the High Accuracy model in Guppy 6.0.7 (https://community.nanoporetech.com) with Qscore limit set to nine and min read length to 200 bp.

All bioinformatic analyses were performed using default parameters unless otherwise specified. *De novo* assembly and polishing were performed with Flye 2.6 (6) and Medaka 1.4.4 (https://github.com/nanoporetech/medaka), respectively, and quality assessed with QUAST (http://cab.cc.spbu.ru/quast), resulting in two contigs. The first contig was 7,247 bp long with 1,509× sequencing depth and 43.38% GC content and was similar to *Enterobacterales* plasmids by NCBI-BLAST (7) (ID > 99%; Table 1). The second contig was 4,985,219 bp long with 50× sequencing depth and 52.12% GC content, and corresponded to the chromosome. The tools used for *in silico* speciation, sero- and genotyping, and screening for antibiotic resistance genes (ARG) and the respective results are shown in Table 1. The isolate’s complete identification was *Salmonella enterica* subsp. *enterica* serovar Typhimurium ST19. The MDR phenotype could be explained by the presence of ARGs for beta-lactams and aminoglycosides and mutations conferring resistance to other antibiotics. *In silico* PCR for the presence of prophage ST104, a marker for the pandemic zoonotic MDR *S*. Typhimurium DT104 phage type, was negative (8, 9).

**TABLE 1:** *In silico* characterization of strain Bnaya

Typing parameter	Tool	Reference	Result
Species	KmerFinder 3.2	(10)	*S*. *enterica* subsp. *enterica* (99.14% template coverage)
Serovar	SeqSero2 1.1.0	(11)	Typhimurium (antigen formula, 4:i:1,2)
MLST	MLST 2.0.9 (PubMLST v. 2023–03-13)	(12)	ST19
cgMLST	SalmcgMLST 1.0 (PubMLST v. 2023–03-13)	(12)	cgST47 (2151/2750 matches, 78.2%)
ARG	ResFinder 4.1 MEGAres 3.0	(13, 14)	ARG**:** blaCTX-M-55 (beta-lactamase) aac(6′)-Iaa (aminoglycoside resistance) Mutations**:** *gyr*, *parcC* (fluoroquinolones) *folP* (sulfonamides) *soxS-R* (various antibiotics)
Plasmid	NCBI-BLAST (ID% > 99)	(7)	*E. coli* O22:H8 strain 154 plasmid p3 (99.86/3596/CP067427.1)^*a*^ *S. enterica* subsp. *enterica* strain 3018683606 plasmid pST3606-3 (99.61/3592/CP094335.1)^*a*^ *E*. *coli* Rb-23 plasmid pRb23_4 (99.53/3598/AP025681.1)^*a*^

^*a*^ ID%/accession length/accession n.

In conclusion, the atypical MDR exhibited by strain Bnaya is likely attributed to chromosomal determinants of resistance, which remain to be fully characterized. Further research is needed to identify possible sources of this strain and assess the potential for its spread. These findings underscore the need for ongoing surveillance of antimicrobial resistance in food-producing animals.

## Data Availability

This whole genome project has been deposited in GenBank under the accession number PRJNA944227. The accession number for the genome assembly is CP119866–CP119867 and for the raw reads, it is SRX20065473. The version described in this paper is the first version.
